# A short peptide for efficient cellular mRNA delivery: A potential application for inducing an immune response

**DOI:** 10.1016/j.omtn.2025.102650

**Published:** 2025-07-29

**Authors:** Clémentine Ayélé Teko-Agbo, Emilie Josse, Karidia Konate, Sébastien Deshayes, Pascal de Santa Barbara, Sandrine Faure, Prisca Boisguérin, Eric Vivès

**Affiliations:** 1 PhyMedExp, University of Montpellier, INSERM, CNRS, Montpellier, France; 2 CHU Arnaud de Villeneuve, 371 Avenue du Doyen G. Giraud, Montpellier, France; 3 Bâtiment Institut Universitaire de Recherche Clinique, Cedex 5, 34295 Montpellier, France

**Keywords:** MT: Delivery Strategies, cell-penetrating peptides, nanoparticle, mRNA delivery, vaccine

## Abstract

Nucleic acid molecules are emerging as potential therapeutic tools, as evidenced by the transfection of small interfering RNA (siRNA) molecules in therapeutic applications and messenger RNAs in immunotherapeutic vaccination. In most cases, these nucleic acids are conditioned as lipid nanoparticles made with different lipid moieties to promote their intracellular delivery. Over the past few years, we have documented the delivery of siRNAs using a single short (15 amino acids) peptide called WRAP5, which follows an extremely simplified formulation phase that enables the formation of nanoparticles with a diameter of 60–80 nm. We indeed demonstrated the expected dose-response reduction in the levels of the targeted proteins. To apply this technology to the cellular delivery of mRNAs, we investigated the ability of the WRAP5 peptide to transfect mRNAs of different sizes and promote the expression of their proteins. These peptide-based nanoparticles, which also have diameters ranging from 60 to 80 nm, showed remarkable stability over time when simply stored at 4°C and fully retained their transfection properties *in vitro* for up to several months post-formulation. Interestingly, we demonstrated *in vivo* that these nanoparticles were able to induce an immune response against the protein synthesized from the vectorized mRNA.

## Graphical abstract

**Figure:**
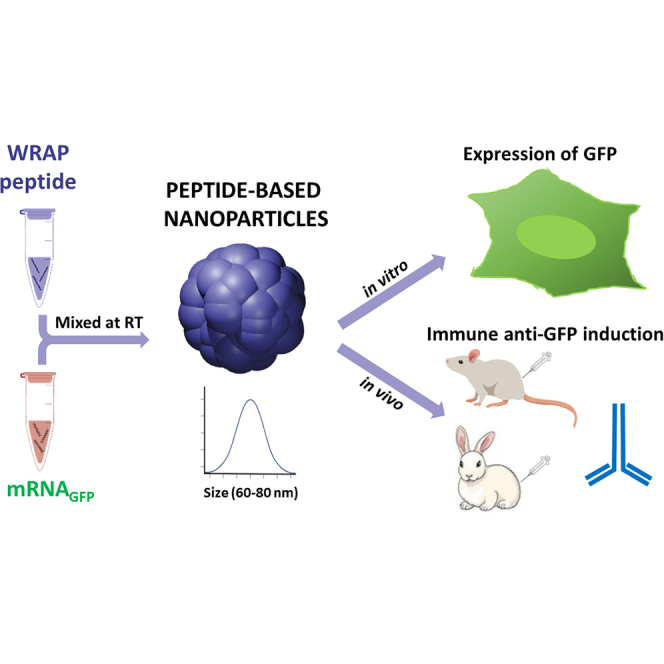

## Teaser

Peptide-based nanoparticle (PBN) technology using one single peptide promotes both cellular delivery of mRNAs and expression of the corresponding protein. These PBNs (60–80 nm) showed stability for up to several months when stored at 4°C and induced an immune response against the protein synthesized from the vectorized mRNA.

## Introduction

Over the past 30 years, numerous peptides called cell-penetrating peptides (CPPs), such as the Tat peptide derived from the Tat protein of HIV1^1^ or the Penetratin peptide derived from the Antennapedia homeodomain,^2^ have been described to be internalized spontaneously into cells and to make it possible to draw various types of covalently linked molecules, such as peptides, proteins, and bioactive molecules, as well as various nucleic acids into the cytoplasm of cells (for recent reviews, see Xu et al. and Boisguérin et al.^3^^,^^4^). A few years ago, we demonstrated the ability of new peptides from the WRAP family (tryptophan [W]- and arginine [R]-rich Amphipathic Peptide) to complex noncovalently with short RNA sequences such as small interfering RNAs (siRNAs) and to form peptide-based nanoparticles (PBNs) with a diameter of approximately 80 nm that are able to transfect these siRNAs into cells.^5^ Once inside the cell, siRNAs allow specific dose-responsive knockdown of the targeted protein, likely through the RNA-induced silencing complex (RISC) system, at levels comparable to those performed by various commercially available cationic lipid transfection reagents.^4^^,^^5^ We also demonstrated that the mechanism of cell uptake of WRAP:siRNA nanoparticles was achieved mainly by direct membrane translocation of the nanoparticle^6^ and that this internalization process had limited cellular toxicity compared with that observed when certain commonly used transfection agents were used.^7^ Indeed, such direct translocation through the plasma membrane could allow it to bypass the endocytose pathway, thus strongly reducing potential nucleic acid degradation.

Six siRNAs have been marketed since 2018, and nearly as many are currently being evaluated in clinical trials.^8^ Five out of six of these therapeutic siRNAs are delivered owing to the direct and covalent attachment of an N-acetyl galactose moiety since these siRNAs are targeted toward the liver.^9^ On the other hand, the remaining siRNAs must be formulated with four different lipid entities (distearylphosphatidylcholine [DSPC], cholesterol, a polyethylene glycol [PEG]-grafted lipid, and proprietary ionizable lipids), a formulation comparable to those found in mRNA vaccines developed against COVID-19.^10^ Amazingly, both lipid nanoparticles (LNPs) formed with either siRNAs or mRNAs were ultimately comparable in size,^5^ with a diameter of approximately 80 nm despite the large difference between one mRNA (approximately 4,000 nucleotides for the SARS-CoV-2 mRNA corresponding to the Spike protein) and one siRNA molecule (42–46 nucleotides).

Since we could efficiently transfect siRNAs with the WRAP5 peptide both *in vitro* and *in vivo*,^5^^,^^11^ we were curious to investigate the possibility of vectorizing mRNAs to obtain the translation of the corresponding protein. Recently, the ability of other cell-penetrating peptides to complex mRNAs and promote cellular internalization has been demonstrated. Nevertheless, these peptides are used either at a low percentage in association with lipids^12^^,^^13^ or in combination with substantial chemical modifications to promote mRNA uptake,^14^^,^^15^ and/or were much longer (30 vs. 15 amino acids for WRAP5).^16^^,^^17^ To date, and to the best of our knowledge, only this latter 30 amino acid peptide has shown the ability to transfect mRNA *in vivo* and induce antibody production.^16^

We first tested the ability of this peptide to autoassemble with mRNAs into stable nanoparticles by screening different charge ratios (CRs) between the number of negative charges carried by the mRNA and the number of positive charges carried by the peptide via a gel retardation assay and dynamic light scattering (DLS) measurements. Using a model mRNA sequence coding for Green Fluorescent Protein (GFP), we effectively expressed the corresponding GFP in HeLa cells, which could be visualized via fluorescence microscopy and quantified via western blot techniques. In addition, these PBNs retained a remarkable transfection capacity over several weeks of storage at 4°C.

Finally, these mRNA-WRAP5 nanoparticles were formulated as concentrated solutions with mRNA concentrations comparable to those in the commercial vaccine solutions marketed by Pfizer/BioNtech, i.e., concentrations of 30 μg of mRNA per 300 μL of injected solution. Notably, the Moderna vaccines contain doses of 100 μg of mRNA per 500 μL of injected solution, and both vaccines showed equivalent efficacy.^18^ In the same way as in the Moderna and Pfizer vaccines, we integrated PEG moieties into the nanoparticles for *in vivo* application, as peptide chemistry allows N-terminal PEG grafting to the WRAP peptide. Formulated mRNA-loaded nanoparticles containing 5% PEGylated WRAP were also characterized in terms of size and stability over time. These PEGylated PBNs were subsequently injected into the forelimbs of chicken embryos to verify GFP expression *in vivo*. Finally, we injected PEGylated PBNs containing 5 μg and 15 μg of mRNA into mice and rabbits, respectively, via a series of intramuscular (IM) injections.

In conclusion, this simplified process for formulating mRNA preparations based on the use of a single ingredient, namely, the WRAP5 peptide, makes the preparation of mRNA nanoparticles much easier, greatly reduces transport and storage constraints at low or ultralow temperatures, and keeps them active over an extended period of several weeks upon simple transportation and/or storage at 4°C, potentially opening up the prospect of wider access to mRNA transfection technology.

## Results

### Determination of the optimal WRAP5:mRNA_GFP_ formulation for cellular GFP expression

**Figure 1:**
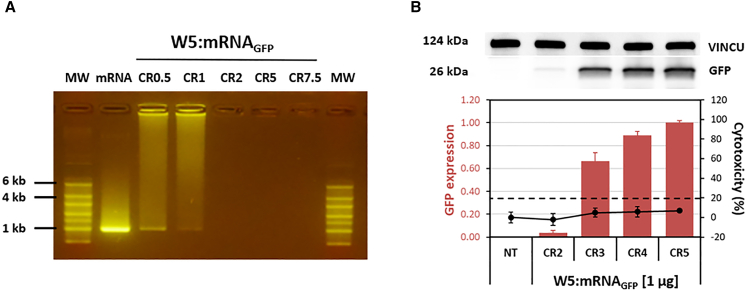
Complex formation of WRAP:mRNA_GFP_ (A) Gel retardation assay of 1 μg of mRNA_GFP_ (996 nt) with different charge ratios (CR0.5, CR1, CR2, CR5, and CR7.5) of the WRAP peptide. Lanes 1 and 8 were loaded with a standard mRNA ladder (Thermo Scientific SM1821). (B) WRAP5:mRNA_GFP_ nanoparticles at different charge ratios (CR2, CR3, CR4, and CR5) encapsulating 1 μg of mRNA_GFP_ (996 nt) were transfected directly after formulation into HeLa cells. After 24 h, GFP expression was revealed by western blot, which revealed CR-dependent GFP expression starting with the lowest CR of 2. The signal intensities of the GFP bands were normalized to those of the corresponding VINCULIN (VINCU) bands. The threshold for a nontoxic condition (= 20%) is visualized by a dashed line. The data represent the means ± SDs of *n* = 2 independent experiments (each in duplicate).

To perform the transfection of mRNA into cells, we first determined the optimal formulation conditions between the WRAP peptide as a carrier and the mRNA encoding the green fluorescent protein (mRNA_GFP_). Therefore, we defined the optimal charge ratio (CR) by calculating the number of anionic charges carried by mRNA_GFP_ (996 nucleotides), which corresponds to the number of phosphate groups per mol of mRNA and the number of cationic charges carried by the peptide. The latter was defined as the total number of cationic charges per mol of the WRAP5 peptide, specifically five charges derived from the terminal α-amino group and four arginine residues. The C-terminal end of the peptide remained neutral due to the presence of an amide group. We thus incubated the mRNA_GFP_ (1 μg) with the peptide at different CRs (0.5, 1, 2, 5, and 7.5). For instance, three nanomoles only (6.3 μg) are sufficient to complex 1 μg of mRNA at a CR5 ratio. We first applied the complexes on an agarose gel to evaluate the complexation of mRNA_GFP_ with the WRAP peptide (Figure 1A). We observed that for low charge ratios (CR0.5 and CR1), some mRNA_GFP_ molecules did not fully complex with the peptide and still migrated within the gel (trail-shaped band). From CR2 and higher, the peptide induced the complete blockade of mRNA mobility within the gel, demonstrating that all the mRNA_GFP_ molecules were complexed with the WRAP5 peptide.

We then confirmed GFP expression after incubating HeLa cells with nanoparticles at a charge ratio that demonstrated complete complexation of the mRNA_GFP_ (Figure 1B). Using 1 μg of mRNA_GFP_, we observed an increasing level of GFP expression between CR3 and CR5 (Figure 1B). For these charge ratios (CR2 to CR5), we did not observe any toxicity as measured by the lactate dehydrogenase (LDH) assay.

For higher mRNA_GFP_ amounts (2 μg), the levels of GFP expression at CR2 to CR5 were similar to those obtained for 1 μg but with greater cytotoxicity, increasing to 40% (Figure S1). Therefore, we selected a charge ratio of 5 with 1 μg of mRNA_GFP_ to perform the assays described in the next sections.

Next, we investigated the physical characteristics of the formulated nanoparticles at different CRs ranging from CR2 to CR5 via dynamic light scattering (DLS). In all cases, measurements performed directly after the formulation of the nanoparticles revealed mean diameters ranging from 53 nm to 95 nm (see Table 1) and polydispersity index (PdI) values ranging from approximately 0.3 to 0.4. Once the nanoparticles were formulated, we did not observe an important variation in diameter over time, up to 2 weeks (d14), upon storage at 4°C, except for the CR2 nanoparticles (see Table 1; Figure S2).

**Table 1:** Dynamic light scattering characterization of the WRAP:mRNA nanoparticles depending on the charge ratio (CR)

Condition	Time	Z-average (nm)	PdI
CR2	d0	95.1 ± 18.2	0.293 ± 0.040
	d7	94.1 ± 27.5	0.245 ± 0.010
	d14	163.8 ± 79.6	0.269 ± 0.093
	d30	ND	ND
CR3	d0	84.4 ± 22.1	0.342 ± 0.054
	d7	ND	ND
	d14	97.1 ± 21.3	0.338 ± 0.035
	d30	ND	ND
CR4	d0	84.7 ± 141	0.399 ± 0.006
	d7	ND	ND
	d14	92.2 ± 8.4	0.348 ± 0.008
	d30	ND	ND
CR5	d0	53.9 ± 8.8	0.361 ± 0.024
	d7	52.4 ± 9.4	0.340 ± 0.051
	d14	84.6 ± 4.3	0.336 ± 0.045
	d30	73.6 ± 11.6	0.357 ± 0.069

The WRAP:mRNA_GFP_ complexes were formed at the indicated CR using 1 μg of mRNA_GFP_ (996 nt) in an aqueous solution of 5% glucose for mean size (Z-average) and polydispersity index (PdI) acquisition. *n* ≥ 2 independent formulations (three measures per run). ND, not determined. The different formulations were analyzed 30 min after their preparation (d0) and 7, 14, and 30 days (d7, d14, and d30, respectively) after their preparation and storage at 4°C between the measurements.

The smallest WRAP5:mRNA_GFP_ nanoparticles were observed with a charge ratio of 5. Moreover, this condition revealed apparent long-term stability at 4°C (up to three months, d90, Table 1; Figures S2D and S4), which is rather interesting for commercialized mRNA vaccines stored at very low temperatures (−80°C to −20°C) and their sensitivity to low (4°C) or ambient temperatures.^19^^,^^20^

**Figure 2:**
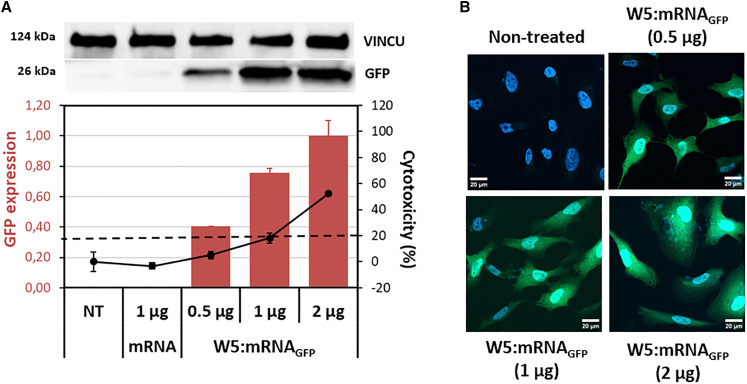
WRAP5:mRNA_GFP_ nanoparticles induced GFP expression in a dose-dependent manner WRAP5:mRNA_GFP_ nanoparticles at CR5 encapsulating different amounts of mRNA_GFP_ (996 nt), 0.5, 1, or 2 μg, were transfected 1 day after formulation into HeLa cells. (A) After 24 h, GFP expression was revealed by western blot, which revealed dose-dependent GFP expression starting with the lowest amount of 0.5 μg. The signal intensities of the GFP bands were normalized to those of the corresponding VINCULIN (VINCU) bands. The threshold for a nontoxic condition (= 20%) is visualized by a dashed line. The data represent the means ± SDs of *n* = 2 independent experiments (each in duplicate). (B) Representative images of confocal microscopy images of WRAP:mRNA_GFP_ nanoparticles (CR5) at 0.5 μg, 1 μg, and 2 μg compared with nontreated HeLa cells. After 24 h of incubation, the cells were fixed and imaged: green = GFP expression and blue = Hoechst dye for nuclear labeling. White bar = 20 μm.

We then evaluated whether the quantity of transfected mRNA could influence the formation of PBNs and/or the transfection rate. For this purpose, we chose three different amounts of mRNA, namely, 0.5, 1, and 2 μg. These samples of mRNA_GFP_ were all formulated as nanoparticles with the WRAP5 peptide at CR5 (Figure 2A). The day after their formulation, the HeLa cells were transfected, and we observed an increase in the GFP level with increasing mRNA-GFP quantity 24 h post-transfection, which was likely correlated with increasing mRNA amounts. This finding was confirmed by confocal microscopy, which revealed that increased GFP intensity was correlated with increased mRNA expression (Figure 2B). However, we also observed greater toxicity, probably due to an excess of free WRAP5 peptide when 2 μg of mRNA was transfected.

Finally, we evaluated whether the presence of serum interfered with the transfection efficiency. Indeed, during the transfection process, the use of serum, regardless of the nature of the transfection reagents, is commonly not recommended, as mentioned by most suppliers of commercially available nucleic acid carriers. We thus reproduced the same transfection conditions but with a standard 10% serum content in the culture medium. By confocal microscopy, we observed a lower level of transfection in the presence of serum than in the absence of serum, with nearly no GFP expression (Figure S3). This reduction is likely due to destabilization of the PBNs by serum proteins or interference of serum proteins impairing the interactions of the PBNs with the plasma membrane.

### WRAP5:mRNA_GFP_ nanoparticles with increased stability

**Figure 3:**
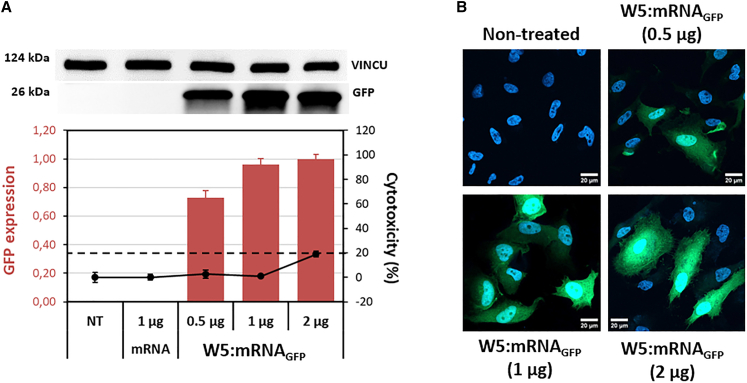
WRAP5:mRNA_GFP_ nanoparticles maintained GFP expression after a 4°C storage period of 30 days (A) WRAP5:mRNA_GFP_ nanoparticles at a charge ratio of 5 (CR5) encapsulating different amounts of mRNA_GFP_ (0.5, 1, and 2 μg, 996 nt) were transfected into HeLa cells after a 4°C storage period of 30 days. After 24 h, GFP expression was detected via western blotting. The threshold for a nontoxic condition (= 20%) is visualized by a dashed line. The signal intensities of the GFP bands were normalized to those of the corresponding vinculin (VINCU) bands. The data represent the means ± SDs of *n* = 2 independent experiments (each in duplicate). (B) Representative images of confocal microscopy images of WRAP:mRNA_GFP_ nanoparticles (CR5) at 0.5, 1, and 2 μg compared with nontreated HeLa cells after a storage period of 30 days. After 24 h of incubation, the cells were fixed and imaged: green = GFP expression and blue = Hoechst dye for nuclear labeling. White bar = 20 μm.

We evaluated the physical characterization of PBNs via DLS and observed the stability of the formed nanoparticles over 1 month (Figure S2D). We also verified the transfection efficacy of these nanoparticles after such a long period. We proceeded in the same way and observed high expression of the GFP in a dose-dependent manner, starting with the lowest amount of 0.5 μg of transfected mRNA (Figure 3A), 30 days after formulation and storage at 4°C. Fluorescence microscopy also revealed dose-responsive staining of the cells at 30 days post-formulation (Figure 3B). We also observed a significant increase in the rate of mRNA_GFP_ transfection using nanoparticles that were formulated 90 days before transfection (Figure S4). Surprisingly, the measured cytotoxicity (Figures 3A and S4) was very weak at 0.5, 1, and 2 μg of transfected mRNA, whereas freshly prepared nanoparticles showed some cytotoxicity (∼20%) at a mRNA_GFP_ concentration of 2 μg (Figure S1).

### Influence of mRNA length on WRAP5-based nanoparticle formation

We also aimed to assess whether the size of the vectorized mRNA affects PBN formation and the corresponding transfection efficiency. To avoid any potential sequence-dependent effects by using different mRNAs coding for various proteins, we constructed three mRNAs, each of which was based on a repeated single mRNA sequence coding for GFP (overall size of 1,200 nt). The longer sequences (2,643 nt and 4,803 nt) also contained the first open reading frame (ORF), which led to the effective translation of a single copy of the GFP, combined with additional noncoding sequences (two mock sequences for the 2,643-nt-long sequence and five mock sequences for the 4,803-nt-long sequence; see Table S3; Figure S5), in which the active start codon was mutated. In other words, for all the constructs, only the first ORF induced GFP expression. This finding also confirmed that the level of the GFP across the three sequences was expected to be identical. All the mRNA sequences also contained one 5′ cap sequence and one 3′ UTR-polyA sequence (overall length of 480 nt), which is usually and commonly integrated into vaccine mRNAs. In addition, we compared both PBN formation via these different mRNA constructs and their relative transfection rates.

The preparation mode of the nanoparticles made with these three mRNA_GFPs_ was exactly the same as that adopted for the initial mRNA_GFP_ (996 nt). We standardized the PBN formulation for these three new mRNAs via a CR5. We first observed a slight increase (approximately 15%) in the mean size of the two longer mRNAs, the 2,643-nt-long and 4,803-nt-long mRNAs, compared with the parent 1,200-nt-long mRNAs (see Table 2; Figure S6). Since we formulated the same amount (1 μg) of each mRNA, there were obviously fewer mRNA copies within PBNs made with longer mRNAs. Again, we observed rather good stability of the formed PBNs over time (21 days of storage at 4°C), with a mean size maintained below 90 nm for all formulations (Table 2). The PdI values were also determined to be approximately 0.400 for all the PBNs (Table 2).

**Table 2:** Dynamic light scattering characterization of the WRAP:mRNA nanoparticle depending on the mRNA length

Condition	mRNA_GFP_ [nt]	Z-average [nm]	PdI
W5:mRNA_GFP_ [d0]	996	56.4 ± 7.5	0.383 ± 0.031
	1,200	60.7 ± 5.6	0.443 ± 0.054
	2,643	72.5 ± 9.7	0.429 ± 0.020
	4,803	74.0 ± 9.9	0.409 ± 0.001
W5:mRNA_GFP_ [d21]	996	72.4 ± 2.3	0.351 ± 0.003
	1,200	68.3 ± 7.3	0.431 ± 0.078
	2,643	75.7 ± 0.5	0.380 ± 0.036
	4,803	84.0 ± 9.4	0.440 ± 0.089

WRAP:mRNA_GFP_ complexes were formed at a charge ratio of 5 (CR5) using 1 μg of mRNA of different lengths (996 nt, 1,200 nt, 2,643 nt, and 4,803 nt) in an aqueous solution of 5% glucose for mean size (Z-average) and polydispersity index (PdI) acquisition. *n* ≥ 2 independent formulations (three measures per run).

**Figure 4:**
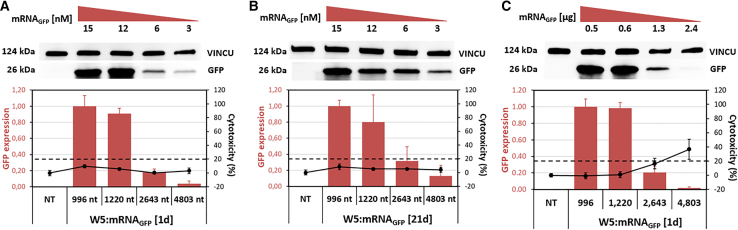
WRAP5:mRNA_GFP_ nanoparticles with different mRNA lengths presented different levels of protein expression WRAP5:mRNA_GFP_ nanoparticles at CR5 encapsulating 1 μg mRNA_GFP_ of different lengths (996 nt, 1,200 nt, 2,643 nt, and 4,803 nt) were transfected into HeLa cells 1 day after formulation (A) or after a storage period of 21 days at 4°C (B). WRAP5:mRNA_GFP_ nanoparticles at CR5 encapsulating an equivalent 7.5-nM concentration of mRNA_GFP_ with different lengths (996 nt, 1,200 nt, 2,643 nt, and 4,803 nt) were transfected into HeLa cells on the day of their formulation (C). Twenty-four hours after transfection, GFP expression was detected via western blotting. The threshold for a nontoxic condition (= 20%) is visualized by a dashed line. The signal intensities of the GFP bands were normalized to those of the corresponding vinculin (VINCU) bands. The data represent the means ± SDs of *n* = 2 independent experiments (each in duplicate).

When these nanoparticles were evaluated for their transfection efficiency and ability to induce GFP expression, we observed a direct correlation between the molar quantity of mRNA encapsulated within the nanoparticles and the resulting GFP expression level. As previously mentioned, a fixed mRNA mass (1 μg) was used to formulate the WRAP:mRNA_GFP_ nanoparticles. Given that the molar quantity of mRNA is inversely proportional to its length, a 4-fold increase in size results in a 4-fold decrease in molar quantity for the same mass. For example, when comparing a 1,200-nt mRNA with a 4,800-nt mRNA, the latter is present in approximately four times fewer copies at an equivalent mass of 1 μg. Consequently, a reduction in GFP levels was observed when fewer mRNA copies were delivered to the cells, both at d1 and d21 post-formulation (Figures 4A and 4B).

When we adjusted the mRNA mass to obtain the same molar quantity (Figure 4C), we observed the same reduction in the GFP level as that recorded above with the same mass of 1 μg. The expression of GFP was much lower for the 4,803-nt-long mRNA than for the 1,220-nt-long mRNA. Even if some reduction in GFP expression was also induced by some toxicity (almost 40%) when the mRNA quantity was high, it thus appeared that the transfection of the large 4,803-nt mRNA appeared to be less potent than that observed for the smaller mRNAs (996 or 1,200 nt), despite a rather close nanoparticle size and distribution. This aspect will require additional evaluations with another set of mRNAs of different lengths.

### PEGylation of the WRAP5:mRNA_GFP_ nanoparticle for *in vivo* development

With the prospect of injecting a vaccine amount of mRNA, we first verified PBN formation at a concentration corresponding to that currently used in commercial mRNA-based vaccines. For example, the Moderna vaccine contains 100 μg of mRNA injected in a 500-μL total volume, whereas the injected dose of the BioNTech vaccine is lower, with 30 μg of mRNA injected in a 300-μL volume. We thus tested an equivalent concentration of 10 μg of mRNA per 100 μL of solution under our experimental conditions. This mRNA concentration is 20-fold greater than the concentration used in our previous formulations and transfection experiments. Concerning the improvement in biodisponibility and biodistribution observed when WRAP:siRNAs_GFP_ nanoparticles were injected *in vivo,*^21^ we formulated our concentrated formulations of nanoparticles by introducing 5% of the PEGylated form of the WRAP peptide out of the normal version of the WRAP peptide. This percentage corresponded to an intermediate value of PEGylated lipids introduced in the Moderna and BioNTech vaccines (from 3% to 6%, depending on the manufacturer). During nanoparticle formation, we expected the highly hydrophilic character of the PEG moiety to be exposed at the nanoparticle surface. We previously reported that a low percentage of PEGylated moieties (up to 20%) did not impair the effective and efficient transfection of siRNAs.^21^ Conversely, when a greater proportion was integrated into these nanoparticles, we clearly showed a reduction in the transfection efficacy, likely because steric hindrance of the PEG chains between the PBNs and the cell membrane prevents the fusion process from being triggered.^21^

First, we evaluated the transfection potency of PEGylated WRAP PBNs in HeLa cells via 1%, 5%, and 10% PEGylation compared with that of naked nanoparticles (Figure S6). All the formulated mRNA_GFP_-loaded PBNs were revealed to have the same transfection capacity.

Then, we controlled the ability of this highly concentrated mixture of 95% WRAP and 5% PEG-WRAP peptides to form nanoparticles in the presence of mRNA_GFP_. We clearly observed the spontaneous formation of PBNs over a 30-min period after mixing the peptides and the mRNA (Figure S6A), as characterized for the diluted preparations (Table 1). By DLS, we sometimes observe heterogeneity in the PBN population. We determined that short (5 min) ultrasound treatment could restore the homogeneity of the nanoparticle population. Thus, we showed, in general, an identical mean size (in the range between 50 and 80 nm) to that of the “diluted” formulations but with significantly lower polydispersity indexes (<0.200), particularly for the PEG-containing nanoparticles (Figure S6C). These nanoparticles were then evaluated regularly for their stability throughout storage at 4°C, as performed with the diluted forms. Again, we noticed excellent stability once the nanoparticles were formed, which occurred over 48 days. In addition, the transfection of the mRNA was clearly effective at 48 days post-formulation (Figure S6B).

**Figure 5:**
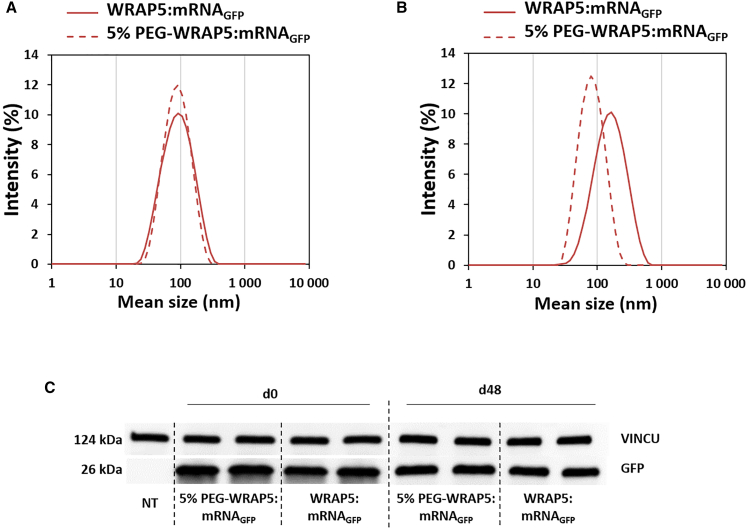
Evaluation of PEGylated WRAP:mRNAGFP nanoparticles compared with naked nanoparticles WRAP5:mRNA_GFP_ or 5% PEG-WRAP5:mRNA_GFP_ nanoparticles at a charge ratio of 5 (CR5) with 1 μg of mRNA_GFP_ (996 nt) were measured via DLS after 1 day (d1) (A) and 48 days (d21) (B) after formulation. For the measurements, the nanoparticles were stored at 4°C. *n* ≥ 2 independent formulations (three measures per run). (C) HeLa cells were transfected with concentrated WRAP5:mRNA_GFP_ or PEG-WRAP5:mRNA_GFP_ nanoparticles at CR5 encapsulating 1 μg of mRNA_GFP_ the day of their formulation (left panel, d0) or after 48 days of storage at 4°C (right panel, d48). After 24 h, GFP expression was detected via western blotting as previously described. The experiment was performed in duplicate. The signal intensities of the GFP bands were normalized to those of the corresponding vinculin (VINCU) bands.

We also verified that the transfection rate was equivalent regardless of whether the nanoparticles contained 5% PEG-WRAP moieties. Transfections were identically performed in parallel and in duplicate with nanoparticles formed with 100% WRAP peptide (WRAP5:mRNA_GFP_) or with 95% WRAP/5% PEG-WRAP5 peptides (PEG-WRAP5:mRNA_GFP_). As shown in Figure 5, we did not observe any significant differences in the level of translated GFP between the two forms of nanoparticles. We also verified the ability of these PBNs to transfect cells after a long storage period at 4°C. Both PBN types, with or without the PEG moiety, retain their ability to transfect cells after 48 days (d48) of storage (Figure 5).

### Evaluation of GFP expression in a chick embryo model using WRAP:mRNA nanoparticles

To assess the capacity of WRAP:mRNA nanoparticles to enable *in vivo* GFP expression, we selected the chick embryo as a model. This system has been widely used to modulate gene expression during limb development.^22^^,^^23^ However, the use of PBNs for mRNA transfection has not been reported in this context. We formulated nanoparticles incorporating 5% of the PEGylated form of the WRAP5 peptide into the standard WRAP peptide to enhance *in vivo* stability.^11^^,^^21^ mRNA_GFP_-loaded 5% PEG-WRAP5 nanoparticles were directly injected into the forelimb buds of chick embryos at Hamburger and Hamilton (HH) stages 28–29, corresponding to embryonic day 6 (E6).^24^ The development of the chick embryo was allowed for 24 h until E7 (HH stage 31).

To improve the visualization of GFP-positive cells, we modified and optimized the RapiClear tissue-clearing protocol, combining it with GFP immunofluorescence detection. Using this technique, we observed that only the WRAP5:mRNA_GFP_-treated forelimbs contained GFP-positive cells. Furthermore, confocal imaging (z-stack acquisition) revealed GFP-positive staining in mesenchymal-derived cells located in the medial part of the forelimb (Figure S7). This demonstrated the ability of WRAP5:mRNA_GFP_ to drive rapidly expression of GFP in muscle tissues *in vivo*.

### WRAP5:mRNA_GFP_ nanoparticle-induced antibody production *in vivo*

We then evaluated whether such PBNs were able to trigger an immunological response following IM injection of 5 μg or 15 μg of mRNA_GFP_ in mice (*n* = 5) and rabbits (*n* = 2), respectively. Preimmune serum was collected from the animals just before the first injection on day 0. WRAP5:mRNA_GFP_ solutions were formulated at an mRNA concentration (1 μg of RNA in a 10 μL volume) identical to that used for commercial doses of the anti-COVID-19 vaccine. Therefore, WRAP5:mRNA_GFP_ nanoparticles were formulated containing 5 μg of mRNA_GFP_ in 50 μL and 15 μg of mRNA_GFP_ in 150 μL of solution for administration to the rear legs of five mice and two rabbits, respectively. WRAP:mRNA_GFP_ nanoparticles were injected IM in the 5% glucose solution we used for the nanoparticle formulation without any immunostimulatory adjuvants. The mice were injected again at days 14 and 28, and the rabbits were injected again at days 7, 14, and 34 with the same solution initially prepared and stored at 4°C throughout the entire vaccination protocol. Intermediate blood collection was performed on the mice and rabbits at days 25 and 28, respectively. The animals were then euthanized, and total blood was collected from the mice and rabbits at days 35 and 42, respectively.

**Figure 6:**
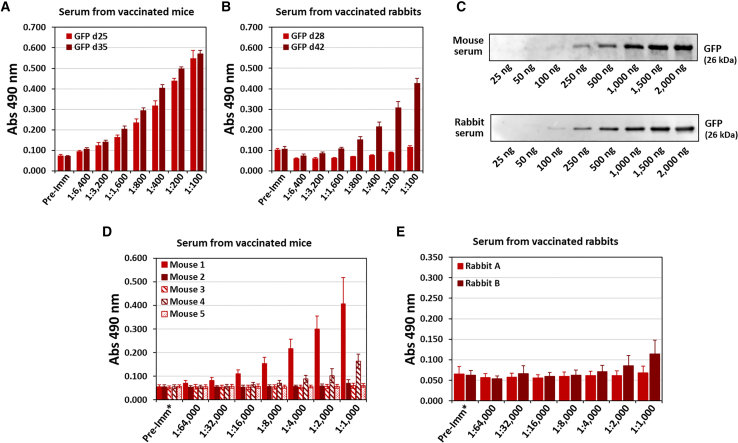
Determination of anti-GFP titers in mouse and rabbit serum by ELISA and western blot Recombinant GFP (50 ng) was coated in ELISA wells, and detection was then performed by using diluted serum (from 1:100 to 1:20,000) from either mice (A) or rabbits (B). Serum samples were collected on day 25 (d25, light red) and day 35 (d35, dark red) from the mice or on day 28 (d28, light red) and day 42 (d42, dark red) from the rabbits. (C) Detection of the recombinant GFP by western blot using whole serum (dilution 1:1,000) from mice (lower panel) or rabbits (upper panel). The mass values indicate the quantity of loaded recombinant protein. (D) Comparison of the anti-GFP antibody titers (from dilutions of 1:1,000 to 1:64,000) in each individual mouse (from mouse 1 to mouse 5). (E) Comparison of the anti-GFP antibody titers (from dilutions of 1:1,000 to 1:64,000) in each individual rabbit (rabbit A and rabbit B).

We then analyzed whether anti-GFP-specific antibodies could be detected by ELISA in mouse and rabbit sera. Interestingly, we observed significant dose-responsive detection of polyclonal antibodies issued from the serum mixture from both species (Figure 6A). Indeed, no response was recorded when the preimmune sera from both species were incubated at a 1:100 dilution. We detected slightly more anti-GFP antibodies in the mice than in the rabbits (Figures 6A and 6B). Interestingly, we also reported that the titers of anti-GFP antibodies were greater after the last injection of PBNs when we compared the first and second collected sera at d25 and d35 for the mice and d28 and d42 for rabbits, respectively (Figures 6A and 6B). It is likely that the last injection significantly increased the immunogenic response in both species. In addition, the same initial PBN preparation, formulated at the beginning of the immunization campaign, was used from the first to the last injection in all the animals. The mRNA formulation was simply stored at 4°C for 5 weeks. Since we clearly observed an increase in antibody induction, this confirmed the stability of these PBNs not only in terms of physical stability but also in terms of mRNA transfection, leading to an increase in the immunological response, as previously shown in our *in vitro* experiments (Figures 3, 4, and 5).

To further confirm the specificity of the ELISA response against the GFP, we performed a western blot characterization of the obtained serum. We first loaded increasing amounts of purified recombinant GFP (ranging from 25 ng to 2 μg) onto an SDS-PAGE gel. Next, we used a 1:1,000 dilution of either mouse or rabbit serum to detect the GFP band on the membrane. In both species, the recombinant GFP was clearly detected, starting from 100 ng with mouse serum and 250 ng with rabbit serum (Figure 6C).

We also wanted to evaluate the level of immunological response in each individual animal. We performed a new series of ELISAs using each of the mouse and rabbit sera diluted from 1:1,000 to 1:64,000 (Figures 6D and 6E). Indeed, one of the five mice (mouse 1) highly responded with GFP antibody detection down to a 1:64,000 dilution. A second mouse (mouse 4) responded weakly with GFP antibody detection only at a 1:8,000 dilution and higher. The other three mice (mice 2, 3, and 5) did not show any immunological response. This result was disappointing since all the mice received the same injections from the same WRAP:mRNA_GFP_ formulation at days 0, 14, and 28. However, such variability in the immune response upon an equivalent immunization procedure has already been reported.^25^ Such variation in immunological response was also found in rabbits, with one out of the two rabbits without any immune response (Figure 6E).

We also examined whether the WRAP5 peptide elicits an immune response by assessing the production of anti-WRAP5 antibodies. This evaluation is crucial, as an immune response against WRAP5 could compromise its effectiveness as a delivery agent, particularly in applications requiring repeated administrations, such as vaccination protocols. Given its short length of only 15 amino acids, WRAP5 might be considered too small to trigger an immune response. It is generally recognized that peptides of this size typically require conjugation to a larger carrier protein (e.g., BSA or KLH) to induce antibody production upon injection. However, since the WRAP5 peptide is formulated as a nanoparticle, its immunogenic potential remains a concern. To investigate this, we coated ELISA plates with 200 ng of pure WRAP5 and incubated them with serum from both mice and rabbits, followed by detection with horseradish peroxidase (HRP)-conjugated anti-mouse or anti-rabbit secondary antibodies. No significant signal was observed above background levels (Figure S8). These findings suggest that WRAP5 does not provoke a detectable humoral immune response, supporting its suitability for use in repeated mRNA delivery applications, including vaccination strategies.

## Discussion

A few years ago, we developed a 15-amino acid peptide that vectorized into cell-specific siRNA molecules and promoted a reduction in the expression of the targeted protein.^5^ With the development of mRNA-based strategies for vaccination and immunotherapy in cancer treatment, we wanted to investigate the ability of our WRAP technology to vectorize larger nucleic acids than siRNAs, such as mRNAs. In this work, we first clearly observed the formation of nanoparticles with diameters between 60 and 90 nm, with very acceptable polydispersity indexes, which are usually lower than 0.4. Interestingly, we unambiguously monitored the expression of the GFP *in vitro* after the cells were transfected with WRAP5:mRNA_GFP_ in a dose-dependent manner. More importantly, we also observed the expression of the GFP in tissues and increased levels of antibodies against the GFP expressed by the vectorized mRNA_GFP_ *in vivo*, both in mice and rabbits. Thus, in both cases, the formation of WRAP5:mRNA_GFP_ nanoparticles promoted the protection of mRNA against degradation, mainly by RNAs present in different cellular compartments but also in biological fluids. These WRAP5:mRNA_GFP_ PBNs enter the cell, likely by direct translocation through the cell membrane, as we previously reported for the vectorization of siRNAs by the same WRAP5 peptide upon the formation of PBNs.^6^ Compared with the traditional vectorization of siRNAs and mRNAs currently performed with a cocktail of lipid nanoparticles,^10^ it would be interesting to assess whether such direct membrane translocation and the respective decaging of mRNAs in the cytoplasm could contribute to limiting the rate of degradation of the mRNAs all along the endosomal pathway and consequently improving the overall level of mRNA translation.

Another interesting property of these WRAP5:mRNA nanoparticles formulated following a simple mix of a single peptide and the mRNA in a 5% glucose solution at room temperature is that these nanoparticles showed remarkable stability upon simple storage at 4°C, as assessed by the maintenance of their physical nanoparticle structures and, more importantly, by their ability to induce the cellular translation of the mRNA over several weeks or even months. This point is crucial when we examine the recommendations of pharmaceutical companies in the field of mRNA vaccination concerning the limited stability of mRNA lipid nanoparticles at cool or ambient temperatures.^19^^,^^26^

In the field of mRNA transfection, various peptides have already been investigated for their ability to promote the cellular uptake of nucleic acids both *in vitro* and *in vivo*.^12^^,^^13^^,^^14^^,^^15^^,^^16^ Nevertheless, compared with the WRAP5 peptide developed in our study, some of these peptides are associated with lipids,^12^^,^^13^ and another peptide requires the grafting of a stearyl moiety at its N-terminal end.^14^^,^^15^ However, more importantly, these peptides^15^^,^^16^ are much longer (up to 30 amino acids) than the 15-amino-acid-long peptide we designed in our group.^5^^,^^6^^,^^7^ In terms of the immunological response, to the best of our knowledge, only the 30-amino-acid peptide, called RALA, has been shown to be able to transfect mRNAs *in vivo* and induce antibody production.^16^

Thus, in this study, we clearly demonstrated the ability of these WRAP5:mRNA_GFP_ nanoparticles to induce an immunological response *in vivo*, both in mice and rabbits, following a set of injections (three in mice and four in rabbits) throughout a 4- or 5-week period. We unambiguously detected the increase in anti-GFP antibodies in the serum of both species via either ELISA or western blot. Interestingly, again in both species, we detected higher antibody titers in sera collected 1 week after a final intramuscular injection than in sera collected previously after two or three injections in mice or rabbits, respectively. This is likely related to an immunological boost following the latest injection. Notably, the WRAP5:mRNA_GFP_ nanoparticle mixture we used throughout the vaccination protocol was issued from a PBN preparation that was formulated at the beginning of the experiment and then stored at 4°C. Linked to the increase in antibody titers observed after the last injection, this latter information confirms the stability of these nanoparticles over a 4- or 5-week period.

Intriguingly, the animals did not react at the same level, despite having been injected at the same time, three or four times, and again with the same common solution. However, such discrepancies between animals also could be observed in various studies.^16^^,^^27^ Even if we can consider a rather modest immunological response observed in mice and rabbits in this initial trial, we remain convinced that this strategy could be used to develop immune responses, although we are aware that procedures will need to be optimized. Compared with heavy equipment, such as microfluidic devices,^28^ which are required to prepare effective mRNAs-LNPs, the strategy we describe here could offer an alternative strategy to develop preliminary mRNA delivery systems for experimental and/or therapeutic levels based on a simplified formulation protocol and a long-term stability/efficacy period for these nanoparticles. The PBNs we used only contained the WRAP peptide (with or without the PEG moiety), the mRNA_GFP_, and a solution of 5% glucose in pure water. Peptide chemistry allows the design of peptides harboring cell-targeting peptides, which can be grafted onto the WRAP5 peptide to attract these nanoparticles to a specific cell type or tissue. In line with this, we recently developed this technology to target Transferrin receptors or the mitochondrial compartment.^29^^,^^30^ This strategy of transfecting mRNAs into cells could inspire, in the near future, the design of smart PBNs coated with both PEG and a targeting peptide to improve bioavailability while selecting a specific cell type.

Another interesting aspect relies on the ability of these PBNs to increase specific antibody production without any immunostimulatory adjuvants, as admitted for mRNA vaccines using lipid nanoparticles as the delivery system, considering that mRNA-LNP vaccines are immune stimulating per se.^31^ Moreover, the complete immunological profiles of these mRNA lipid nanoparticle vaccines are still not yet completely understood.^32^ Recently, mRNA transfection of secondary lymphatic organs, but not lipid nanoparticle adjustment or RNA expression in muscle, was suspected to be the main driver of the adaptive immune response in mice. Indeed, further investigations of how the immunological response induced by our PBNs is triggered *in vivo* would be interesting.

We also showed that the WRAP5 peptide itself did not provoke a detectable humoral immune response, which could compromise its effectiveness as a delivery agent, particularly in applications requiring repeated administrations, such as vaccination protocols.

However, more research will be imperative to further investigate the behavior of PBNs in an immunological context to develop efficient mRNA vaccines, not only against emerging pathogenic viruses but also to develop efficient and easy-to-handle immunotherapeutic strategies in oncology.

## Materials and methods

### Peptide synthesis

WRAP5 peptide (W5, _H2N-_LLRLLRWWWRLLRLL_-CONH2_) was synthesized via the SynBio3 platform (Institut des Biomolécules Max Mousseron [IBMM], Montpellier) following the Fmoc strategy at a 200-μmole scale as previously described.^5^^,^^7^ The crude products were purified in-house following qualitative analysis by high-performance liquid chromatography (HPLC)/mass spectrometry (MS) (>95% purity). WRAP5 and PEG-WRAP5 (see below) peptides were solubilized in pure water (Sigma-Aldrich, Saint-Quentin-Fallavier, France), and stock solutions (400–1,000 μM) were stored at 4°C.

### Coupling of PEG on the WRAP peptide

The WRAP peptide was assembled on resin as described above. The Fmoc group at the N-terminal end of the peptide (38 μmol) was removed by treating the peptide/resin with a 20% piperidine solution in dimethylformamide (DMF) twice (2 and 15 min). After several washes with DMF, the coupling of the monodisperse Fmoc-NH-PEG(36)-COOH (1897.22 g/mol, purchased from Iris-BioTech, Marktredwitz, Germany) was performed on resin in DMF upon activation of Fmoc-PEG(36)-COOH (146 mg, 77 μmole) using tetramethylfluoroformamidinium hexafluorophosphate (TFFH, 75 mg, 284 μmol). The pH was adjusted to 7.5–8.0 by the addition of 80 μL of N,N-diisopropylethylamine (DIEA) to the reaction tube. The coupling step was carried out for 2 h at room temperature under agitation and monitored via a Kaiser test for residual amine detection. At the end of the coupling step, the Fmoc group of the PEG unit was cleaved via traditional treatment with 20% piperidine in DMF, and the resulting resin was washed five times with 2 mL of DMF, three times with 2 mL of dichloromethane (DCM), and three times with 2 mL of ether. The resin was dried under vacuum for 1 hour. The peptide was then deprotected and cleaved from the resin for 2 h at room temperature by treatment with trifluoroacetic acid (TFA), triisopropylsilane (TIS), and water (92.5%, 5.0%, 2.5%, vol/vol). The crude peptide was precipitated overnight in cold ether, and the mixture was then centrifuged for 5 min at 3,000 rpm. The pellet was resuspended in 30% acetonitrile in water (0.1% TFA), and the H_2_N-PEG(36)-WRAP peptide was purified on a semipreparative HPLC system. The homogeneous fractions containing the expected peptide were pooled and freeze-dried. The peptide was fully characterized by analytical HPLC and electrospray ionization (calculated mass: 3,763.69 g/mol; found mass: 3,764.85 g/mol). The concentration of the purified peptide stock solution was quantified via a NanoDrop instrument by reading the optical density at 280 nm (the extinction coefficient of the WRAP peptide was 16,500 M^−1^ cm^−1^ based on the three tryptophan residues).

### mRNA synthesis

GFP mRNA (sequence in Table S1, supplemental information) was purchased from Tebubio (Le Perray-en-Yvelines, France). All other base editors were produced as follows by TriLink BioTechnologies (San Diego, CA). Briefly, mRNA was transcribed *in vitro* from the PCR product via full substitution of uridine for an analog nucleotide, N_1_-methylpseudouridine. mRNAs were then capped co-transcriptionally via the CleanCap AG analog, resulting in a 5′ Cap 1 structure. *In vitro* transcription was performed, and mRNAs were purified via an RNeasy kit (QIAGEN). The transcribed PCR product included mammalian-optimized UTR sequences and a 120-base poly-A tail. The RNA stock solutions were stored at 1 μg/μL in RNase-free water at −80°C until further use. To evaluate the potential influence of the length of the mRNAs, we designed different mRNAs harboring one common active open reading frame (ORF) for the GFP, with or without an extension of the same GFP sequences, but all were mutated at the AUG start codon (see Figure S5). Therefore, we compared the ability of the same GFP RNA as a single sequence or as a multiple sequence to eliminate sequence dependence during the formulation and transfection steps, on the one hand, and the level of GFP expression, on the other hand, since only one ORF led to the expression of the corresponding GFP.

### Formulations of the mRNA nanoparticles

WRAP5:mRNA nanoparticles were formulated at different charge ratios, CRs, corresponding to the ratio of positively charged amino acids and negatively charged phosphate groups from the nucleic acid. For the cell transfection, which was performed in duplicate in six-well plates with 0.5 μg of mRNA per well with a CR of 5, a total volume of 440 μL of nanoparticle mixture was prepared. A total of 1.1 μL of mRNA (1 μg/μL) was dissolved in 218.9 μL of a 5% (m/v) glucose solution. Based on the number of negative charges present on the mRNA molecule, 5 charge equivalents of a mixture of WRAP5 and PEG-WRAP5 (95% and 5%, respectively) peptides were also dissolved in 5% (m/v) glucose solution in a final 220 μL volume. Both the mRNA and peptide solutions were homogenized, and the mRNA mixture was injected into the peptide mixture. The WRAP5:mRNA solutions were mixed by several successive pipetting steps, allowed to self-assemble for 30 min, and sonicated for 5 min before analysis by dynamic light scattering as described below. The final concentration of mRNA was 2.5 μg/mL.

### Preparation of vaccine formulations

For the preparation of the mRNA nanoparticle solutions dedicated to the mouse and rabbit vaccination processes, we used the same experimental conditions except that the final concentration of mRNA was 1 μg per 10 μL of nanoparticle solution. This final concentration of mRNA corresponded to 100 μg/mL, a concentration 40-fold higher than the concentration used in the *in vitro* experiment described above. For example, to prepare a volume of 100 μL of vaccine solution at a CR of 5, 10 μL of mRNA was dissolved in 40 μL of a 6.25% (m/v) glucose solution (final concentration of 5% glucose). As described above, the mRNA mixture was injected into an equivalent volume (50 μL) of a mixture of WRAP5 and PEG-WRAP5 peptides (95% and 5%, respectively) dissolved in a 5% (m/v) glucose solution. Several successive pipetting steps were applied, and the nanoparticle mixture was left to self-assemble for 30 min. Once prepared, the nanoparticle solutions were stored at 4°C until further use.

### Culture conditions

Human HeLa cells (CCL-2, ATCC), derived from cervical cancer, were grown in complete medium (DMEM with GlutaMAX [Thermo Fisher Scientific, Illkirch, France], 1% penicillin/streptomycin, and 10% fetal bovine serum [FBS] [Sigma-Aldrich, Saint-Quentin-Fallavier, France]) and were maintained in a humidified incubator with 5% CO_2_ at 37°C.

### Gel retardation assay

The capacity of the WRAP peptide to form complexes in the presence of mRNAs was evaluated via a gel retardation assay. Preformed WRAP:mRNA complexes (with a constant mRNA concentration of 1 μg for each condition and WRAP at the indicated charge ratio [CR]) were analyzed by electrophoresis on agarose gels (1%/vol) stained with RiboGreen (Thermo Fisher Scientific, Illkirch, France). Images were acquired, and quantification of the bands was achieved via ImageJ software.

### DLS and zeta potential measurements

The size of the WRAP:mRNA nanoparticles was evaluated via a Zetasizer NanoZS (Malvern Pananalytical, Malvern, UK) in terms of the mean size (Z-average) and particle distribution homogeneity (PdI). Zeta potential was measured in 5% glucose with 2 mM NaCl. All the results were obtained from three independent measurements (three runs for each measurement at room temperature).

### Transfection experiments

HeLa cells were seeded into six-well plates (220,000 cells/well) for western blot analysis and into 12-well plates (110,000 cells/well on a glass cover slide) for confocal imaging, both 24 h before the experiment. For nanoparticle incubation, the cells were incubated with 1,600 μL of fresh prewarmed serum-free DMEM (Thermo Fisher Scientific, Illkirch, France) + 200 μL of the nanoparticle solutions at the indicated concentrations or 200 μL of 5% glucose (m/v) for the nontreated cells. After 4 h of incubation, 200 μL of FBS was added (final FBS concentration = 10%) to each well without removing the transfection reagents. The cells were then incubated for an additional 20 h (total incubation time of 24 h) and finally lysed for western blot evaluation (detailed below). For serum-containing incubations, 200 μL of the nanoparticle solutions were directly added to 1,800 μL of prewarmed DMEM containing 10% FBS.

### Cell cytotoxicity measurement

The cytotoxicity induced by the nanoparticles was evaluated via a Cytotoxicity Detection Kit^Plus^ (LDH, Sigma-Aldrich, Saint-Quentin-Fallavier, France) following the manufacturer’s instructions. At least one well of a six-well plate was used as an LDH-positive control (100% toxicity), and Triton X-100 (Sigma-Aldrich, Saint-Quentin-Fallavier, France) was added at a final concentration of 0.1% (∼15 min incubation at room temperature). Afterward, 50 μL of the supernatant from each well was transferred to a new clear 96-well plate. Fifty microliters of the “dye solution/catalyst” mixture was added to the supernatant, which was subsequently incubated in the dark for 30 min at room temperature. The reaction was stopped by adding 25 μL of HCl (1 N) to each well before the absorption at 490 nm was measured. Relative toxicity (%) = [(exp. value – value nontreated cells)/(value Triton – value nontreated cells)] × 100.

### Western blotting

(1) GFP detection from cell lysate: Transfected cells were washed in PBS and lysed in RIPA buffer (50 mM Tris pH 8.0, 150 mM sodium chloride, 1% Triton X-100, 0.1% SDS [sodium dodecyl sulfate, Sigma-Aldrich, Saint-Quentin-Fallavier, France]) supplemented with protease inhibitors (SigmaFAST, Sigma-Aldrich, Saint-Quentin-Fallavier, France). The cells were incubated for 5 min on ice with 120 μL/well lysis buffer. Thereafter, the cells were scraped and transferred to a 1.5-mL tube. After 5 min on ice, the cell lysates were gently shaken on a rotating wheel in a cold room at 4°C for 15 min and then centrifuged (5 min, 13,500 rpm, 4°C). A total of 115 μL of each supernatant was collected, and protein concentrations were determined in 2 × 20 μL of each supernatant via the Pierce BCA protein assay (Thermo Fisher Scientific, Illkirch, France). The cell extracts were diluted in 2x Laemmli buffer to a final protein concentration of 20 μg and then denatured at 95°C for 10 min.
(2) GFP detection with anti-GFP-serum antibodies: Recombinant GFP (Sino Biological, Eschborn, Germany) was diluted in PBS to a final volume of 15 μL at various concentrations (25 ng, 50 ng, 100 ng, 250 ng, 500 ng, 1,000 ng, 1,500 ng, and 2,000 ng), denatured by adding 15 μL of 2x Laemmli buffer, and then heated at 95°C for 10 min.

In both cases, proteins were separated via a 4%–20% Mini-PROTEAN TGX Precast Gel (Bio-Rad, Marne-La-Coquette, France). After electrophoresis, the samples were transferred onto Trans-Blot Turbo Mini PVDF transfer membranes (Bio-Rad, Marne-La-Coquette, France). The membrane was first saturated with a 5% BSA solution in PBS containing 0.05% Tween. The following antibodies were used: anti-GFP rabbit (Rockland, Tebubio, Le Perray-en-Yvelines, France), anti-GFP serum (produced in this study), anti-vinculin rabbit monoclonal antibody (mAb), anti-mouse immunoglobulin (Ig)G HRP, and anti-rabbit IgG HRP (Cell Signaling, Saint-Cyr-l’Ecole, France). The blots were visualized with Pierce ECL plus western blotting substrate (Thermo Fisher Scientific, Illkirch, France) on an AZURE Imager (Dublin, CA, USA). The signal intensities of the blots were quantified via ImageJ software. Each GFP band intensity of a distinguished condition was then normalized to the corresponding VINCULIN band intensity. Then, we set up the GFP expression scale level between 0 and 1, where 1 represents the higher level of GFP expression and 0 the level of the nontreated cells.

### Chick embryo model, whole-mount clearing analysis, and immunofluorescence analysis

Fertilized regular brown chicken eggs (Les Bruyères, Dangers, France) were incubated at 38°C in a humidified incubator (HB-700-H, Cimuka, Ducatillon, France) until use. Although chick embryo experiments do not require approval by an ethics committee (European law, Article 2016/63/UE), all procedures were conducted in accordance with the ethical guidelines of INSERM and CNRS for animal experimentation. After 2 days of incubation, 4 mL of albumin was removed from each egg, and a small opening was made in the eggshell, which was then sealed with tape to prevent vascularization at the top of the shell. To locate the embryo, eggs were illuminated from below, and a window of approximately 1.5 × 1.5 cm was cut into the eggshell. The eggshell and outer membrane were removed to expose the embryo, which was staged according to the Hamburger and Hamilton (HH) classification^24^ and corresponding embryonic day (E). When the embryos reached HH stages 28–29 (E6), the tape was partially removed to re-expose the embryo and visualize the right forelimb.

A total of 10 μg of mRNA_GFP_ was mixed with 5% PEG-WRAP5 and 95% WRAP5 with a charge ratio of 5 (CR5). Following nanoparticle formation, the solution was stored at 4°C for several days. To facilitate injection visualization, 2 μL of 0.1% Fast Green solution was added to 10 μL of RNA_GFP_-loaded 5% PEG-WRAP5 nanoparticle solution, followed by sonication for 5 min. The solution was loaded into a microcapillary and injected (0.2 μL) into the E6 forelimb under a Leica M165FC stereomicroscope. After injection, the eggs were resealed with fresh tape and incubated for an additional 24 h.

For tissue clearing, E7 forelimbs from both control and mRNA_GFP_-loaded 5% PEG-WRAP5 nanoparticle-injected embryos were fixed at room temperature (RT) on an orbital shaker in 4% paraformaldehyde in PBS for 2 h, followed by a 1-h wash in PBS. The samples were then permeabilized in 2% Triton X-100 in PBS containing 0.05% sodium azide at RT for 4 h with shaking and washed three times in PBS for 15 min each. Blocking was performed at 4°C overnight on an orbital shaker using a solution containing 10% normal donkey serum, 1% Triton X-100, and 0.2% sodium azide in PBS. The samples were subsequently incubated with primary goat anti-GFP antibodies (TEBU, ref. 600-101-215; dilution 1:500) in antibody dilution buffer (1% normal donkey serum, 0.2% Triton X-100, and 0.2% sodium azide in PBS) at 4°C on an orbital shaker for 24 h. The samples were then washed three times in washing buffer (0.2% Triton X-100 in PBS) at RT for 1 h each, followed by an overnight wash at 4°C. Secondary antibody incubation was performed using anti-goat Alexa Fluor 555 (Invitrogen, A21432; dilution 1:300) and TO-PRO-3 for nuclear staining (Invitrogen, T3605; dilution 1:1,000) in dilution buffer at 4°C on an orbital shaker for 24 h. The samples were subsequently washed three times with washing buffer at RT for 1 h each, followed by an overnight wash at 4°C. The final washes were performed with PBS (three times for 15 min each), and the samples were subsequently cleared with RapiClear at RT overnight. The cleared samples were subsequently placed in 2,2′-thiodiethanol solution (Sigma-Aldrich, Saint-Quentin-Fallavier, France) and imaged via a Zeiss LSM780 confocal microscope (×10 or ×20 magnification) via a 488-nm diode laser with a GFP filter (500–542 nm). Image analysis was conducted via Imaris software.

### Confocal microscopy

HeLa cells were transfected as described above (see transfection experiments). After 20 h, the cell nuclei were labeled with Hoechst 33342 (Thermo Fisher Scientific, final concentration of 2 μg/mL) for 10 min. Thereafter, the medium from each well was removed, and the cells were fixed with 2% PFA solution in PBS for 5 min, followed by five washes with PBS and two washes with H_2_O. After the cover slides were dried, they were mounted with homemade Mowiol mounting media. Images of the cells were acquired on an inverted Zeiss LSM800 microscope with an Apo 63x/1.2 W DICIII lens. All confocal acquisitions were performed via the following diode lasers: 405 nm with a Hoechst filter (400–456 nm) and 488 nm with a GFP filter (500–542 nm). Image acquisition was performed sequentially to minimize crosstalk between the fluorophores. Each confocal image was merged and adjusted with the same brightness and contrast parameters via Fiji ImageJ software.

### Immunization protocol in mice and rabbits and serum collection

The immunization protocol was performed by the Biotem Company (Apprieu, France). Five OF1 mice were immunized with three injections of nanoparticle solutions containing 5 μg of mRNA in 50 μL of 5% glucose (m/v). A preimmune serum sample (30–100 μL) was collected from each mouse just before the first injection. Injections were performed at d0, d14, and d28 with the same initially prepared nanoparticle solution. Before each injection, the nanoparticle solution was sonicated for 5 min in a sonication bath. At d25, a first blood collection (corresponding to 30 to 100 μL of serum) was performed, and at d35, the mice were euthanized by exsanguination under anesthesia, and the blood was collected. EDTA (10 μL per mL of blood) was added, and the tubes were gently mixed and stored on ice for 15 min. Total serum (400–600 μL) was then collected after centrifugation (10 min at 2,000 RCF). Serum samples were aliquoted and stored at −20°C until further use.

Two New Zealand White (NZW) rabbits were immunized with four injections of nanoparticle solutions containing 15 μg of mRNA in 150 μL of 5% glucose (m/v). A preimmune serum sample (500–1,000 μL) was collected from each rabbit just before the first nanoparticle injection. Injections were performed at d0, d7, d14, and d34 with the same initially prepared nanoparticle solution. Before injection, the nanoparticle solution was sonicated for 5 min in a sonication bath. At d28, a first blood collection (corresponding to 15–18 mL of serum obtained after centrifugation) was performed, and at d42, rabbits were euthanized as described above, and the whole serum (100–160 mL) was collected after centrifugation. Serum samples were aliquoted and stored at −20°C until further use.

### ELISA determination of anti-GFP antibody titers

A total of 25, 50, or 100 ng of recombinant GFP (Sino Biological, Eschborn, Germany) resuspended in Dulbecco’s phosphate-buffered saline (DPBS) (Corning, Manassas, VA, USA) was used to coat the wells of a 96-well plate at room temperature overnight. The wells were then washed once with DPBS and saturated for 2 h with DPBS containing 2.5% BSA and 0.05% Tween. The wells were washed three times with PBS. Then, the dilutions of the mouse and rabbit sera (from 1:100 to 1:64,000 in PBS containing 0.05% Tween and 0.5% BSA) were incubated in triplicate for 2 h in the different wells. The wells were then washed three times with washing buffer (DPBS and 0.05% Tween) before being incubated for 1 h with an anti-mouse or anti-rabbit HRP-coupled antibody (Cell Signaling, Saint-Cyr-l’Ecole, France). The antibody solutions were discarded, and three additional washes were performed. The wells were then incubated for 20 min with the HRP substrate (250 μL of a 10 mg/mL tetramethylbenzidine solution in DMSO diluted in 25 mL of a 0.05 M Na_2_HPO_4_ and 0.025 M citric acid solution adjusted to pH 5.5 and 80 μL of hydrogen peroxide). The reaction was then stopped upon the addition of 25 μL of a 2 N sulfuric acid solution. The plates were read on a TECAN plate reader (Männedorf, Switzerland) at 490 nm.

## Data availability

The authors confirm that the data supporting the findings of this study are available within the article and/or its supplemental information.

## Acknowledgments

The authors are grateful to Pascal Verdié and Luc Brunel from the SynBio3 platform (Institut des Biomolécules Max Mousseron [IBMM], Montpellier, France) for providing peptide synthesis and peptide purification facilities and to Pierre Sanchez for performing LC-MS analysis. The authors thank Laurie Albuquerque and Florie Lopez from the PhyMedExp Animal Facilities for housing the mice and Chloé Felgerolle for assisting with the animal treatments. This research was funded by the Montpellier University of Excellence Program (MUSE), the 10.13039/501100001665Agence Nationale de la Recherche (ANR-21-CE18-0022-01 to P.B.), and by the 10.13039/501100004794Centre National de la Recherche Scientifique Innovation Program.

## Author contributions

C.A.T.-A., E.J., K.K., P.d.S.B., P.B., and E.V. conducted the experiments. P.B., P.d.S.B., and E.V. designed the experiments and wrote the paper. P.B., P.d.S.B., K.K., S.D., S.F., and E.V. reviewed and edited the manuscript.

## Declaration of interests

The authors declare no competing interests.

## Supplemental information

Document S1. Figures S1–S8 and Tables S1–S4

Document S2. Article plus supplemental information
