# Supplementary material for: A short peptide for efficient cellular mRNA delivery: A potential application for inducing an immune response
Source: Mol Ther Nucleic Acids. 2025 Jul 29;36(3):102650. doi: 10.1016/j.omtn.2025.102650 (PMC12359147; doi:10.1016/j.omtn.2025.102650)
Supplement: Document S1. Figures S1–S8 and Tables S1–S4 [file mmc1.pdf]

OMTN, Volume *36*

Supplemental information

A short peptide for efficient cellular mRNA delivery: A potential application for inducing an immune response

Clémentine Ayélé Teko-Agbo, Emilie Josse, Karidia Konate, Sébastien Deshayes, Pascal de Santa Barbara, Sandrine Faure, Prisca Boisguérin, and Eric Vivès

Table S1: Sequence of the GFP mRNAs

AUGGUGAGCAAGGGCGAGGAGCUGUUCACCGGGGUGGUGCCCAUCCUGGUCGAGCUGGACGGCGACGUAAACGGCCACAA
GUUCAGCGUGUCCGGCGAGGGCGAGGGCGAUGCCACCUACGGCAAGCUGACCCUGAAGUUCAUCUGCACCACCGGCAAGC
UGCCCGUGCCCUGGCCCACCCUCGUGACCACCCUGACCUACGGCGUGCAGUGCUUCAGCCGCUACCCCGACCACAUGAAG
CAGCACGACUUCUUCAAGUCCGCCAUGCCCGAAGGCUACGUCCAGGAGCGCACCAUCUUCUUCAAGGACGACGGCAACUA
CAAGACCCGCGCCGAGGUGAAGUUCGAGGGCGACACCCUGGUGAACCGCAUCGAGCUGAAGGGCAUCGACUUCAAGGAGG
ACGGCAACAUCCUGGGGCACAAGCUGGAGUACAACUACAACAGCCACAACGUCUAUAUCAUGGCCGACAAGCAGAAGAAC
GGCAUCAAGGUGAACUUCAAGAUCCGCCACAACAUCGAGGACGGCAGCGUGCAGCUCGCCGACCACUACCAGCAGAACAC
CCCCAUCGGCGACGGCCCCGUGCUGCUGCCCGACAACCACUACCUGAGCACCCAGUCCGCCCUGAGCAAAGACCCCAACG
AGAAGCGCGAUCACAUGGUCCUGCUGGAGUUCGUGACCGCCGCCGGGAUCACUCUCGGCAUGGACGAGCUGUACAAGUAA

Table S2: Characterization of the WRAP:$mRNA_{GFP}$ nanoparticle depending on the used $mRNA_{GFP}$ amount.

Conditions	Time	$mRNA_{GFP}$ [µg]	Z-Average [nm]	PdI
**W5:$mRNA_{GFP}$ [CR 5]**	d1	0.5	52.5 ± 6.6	0.589 ± 0.236
		1	51.8 ± 6.2	0.383 ± 0.076
		2	66.9 ± 1.0	0.320 ± 0.092
	d30	0.5	67.6± 9.1	0.438 ± 0.017
		1	73.6± 11.6	0.289 ± 0.095
		2	68.7 ± 8.5	0.232 ± 0.029

Footnotes: The WRAP:$mRNA_{GFP}$ complexes were formed at the indicated charge ratio using an mRNA concentration of 0.5 µg, 1 µg or 2 µg in an aqueous solution of 5 % glucose for mean size (Z-average) and polydispersity index (PdI) acquisitions. n ≥ 2 independent formulations (3 measures per run).

Table S3: Sequence length and schematic structure of the 3 GFP mRNAs used.
The different mRNAs were synthesized and provided by Tebubio (Le Perray-en-Yvelines, France).

Target mRNA	eGFP (gene)	UTR + PolyA	Total mRNA length
eGFP	720	480	1,200
eGFP + 2 mock eGFP	2,163	480	2,643
eGFP + 5 mock eGFP	4,323	480	4,803

Table S4: Comparison of WRAP:$mRNA_{GFP}$ nanoparticles with the PEGylated version by DLS.

	d1		d48	
	Z-average (nm)	PdI	Z-average (nm)	PdI
**WRAP5:$mRNA_{GFP}$**	79.8 ± 0.47	0.16 ± 0.01	73.7 ± 0.1	0.16 ± 0.01
**5% PEG-WRAP5:$mRNA_{GFP}$**	79.7 ± 0.31	0.20 ± 0.01	137.7 ± 0.2	0.22 ± 0.01

Footnotes: The WRAP:$mRNA_{GFP}$ complexes were formed at a charge ratio of 5 using an mRNA concentration of 1 µg in an aqueous solution of 5 % glucose for mean size (Z-average) and polydispersity index (PdI) acquisitions. Results were acquired with 3 measures per run.

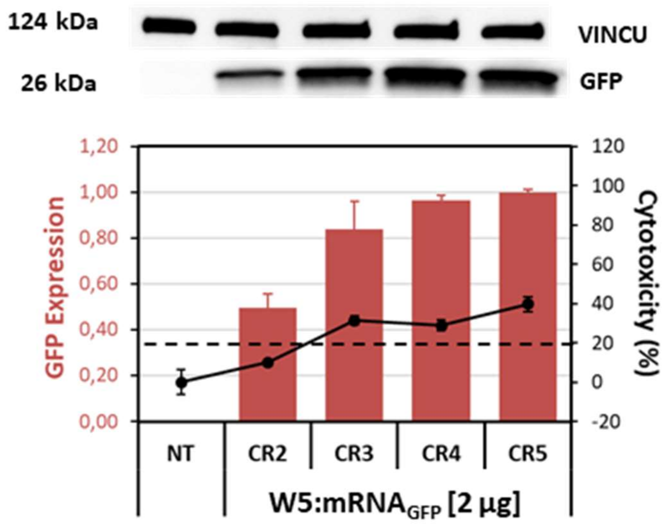

Fig. S1: Complex formation of WRAP:mRNA$_{GFP}$.

WRAP5:mRNA$_{GFP}$ nanoparticles at different charge ratios (CR2, CR3, CR4, and CR5) encapsulating 2 µg mRNA$_{GFP}$ were transfected directly after formulation to HeLa cells. After 24 h, GFP expression was revealed by Western Blot showing a CR-dependent GFP expression starting with the lowest CR of 2. Signal intensities of the GFP bands were normalized to the corresponding Vinculin (VINCU) bands. The threshold for a non-toxic condition (= 20%) is visualized by a dashed line. Data represent the mean ± SD of n=2 independent experiments (each in duplicates).

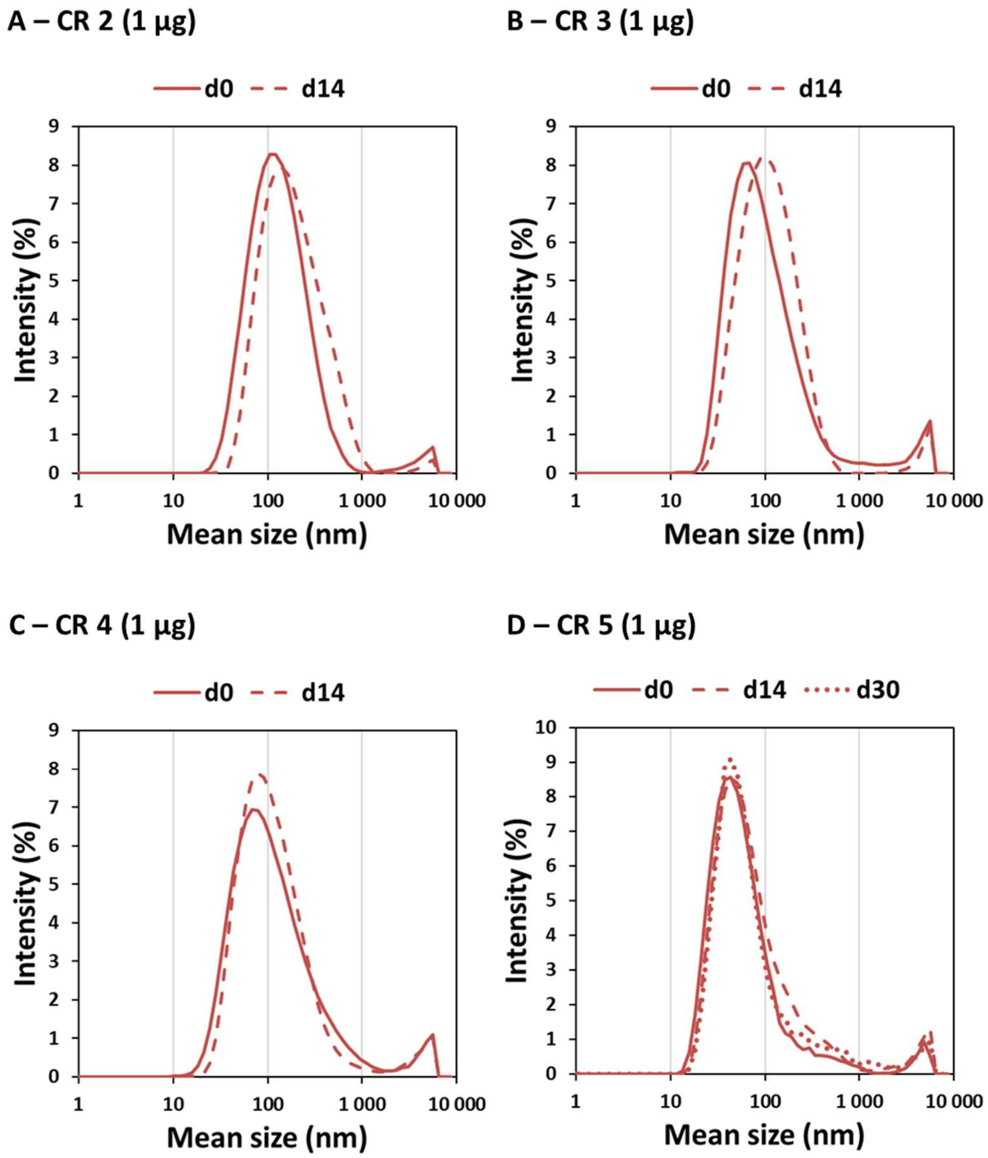

Fig. S2: WRAP5:mGFP nanoparticles measured by DLS. WRAP5:mGFP nanoparticles at different charge ratios (CR 2, CR 3, CR 4, and CR 5) were measured by DLS after formulation (d0) and after 14 days (d14), as well as after 30 days (d30) for CR 5. Data represent the mean of 5 independent experiments (measured with 3 runs each).

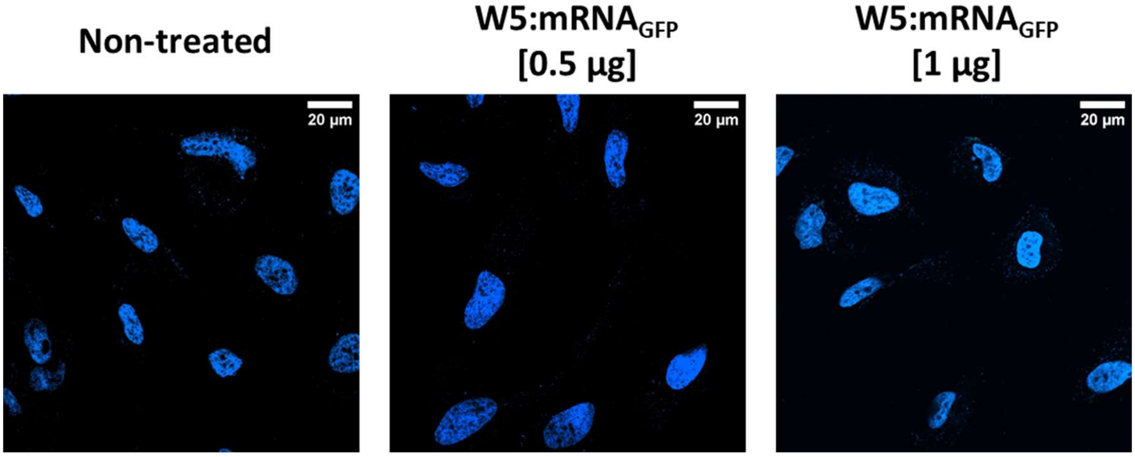

Fig. S3: WRAP5:mRNA$_{GFP}$ nanoparticles do not promote transfection in the presence of serum. Representative images of confocal microscopy acquisitions of WRAP:mRNA$_{GFP}$ nanoparticles (CR5) at 0.5 µg and 1 µg compared to non-treated HeLa cells. After 24 h incubation, cells were fixed and imaged: green = for GFP acquisition and blue = Hoechst dye for nucleus labeling. White bar = 20 µm.

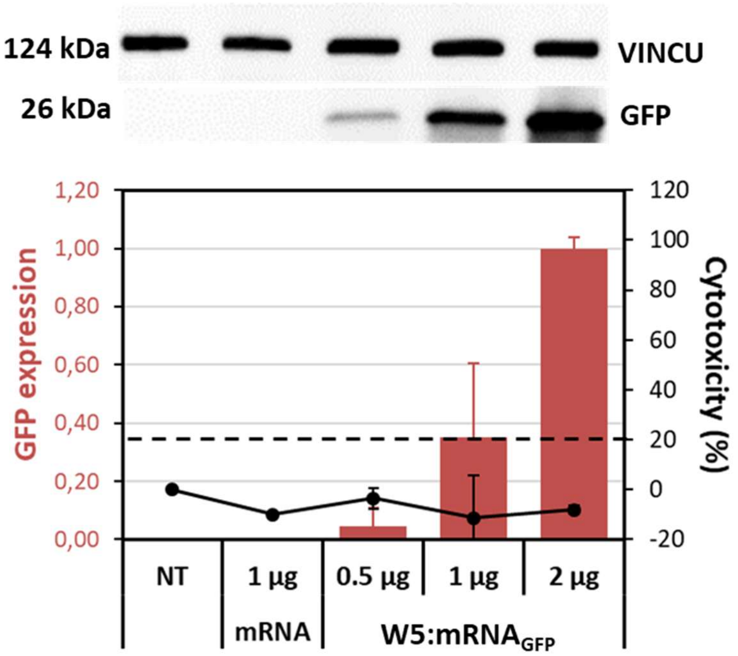

Fig. S4: WRAP5:mGFP nanoparticles transfection efficiency after 90 day-storage. After a 90-day-storage period at 4°C, WRAP5:mRNA$_{GFP}$ nanoparticles at different mRNA amounts (CR5) were transfected to HeLa. GFP expression revealed by Western Blot after a 24 h incubation showed still an important transfection efficiency, even if the relative amount is less important compared to "freshly" formulated nanoparticles. The threshold for non-toxic conditions (= 20%) is visualized by a dashed line. Signal intensities of the GFP bands were normalized to the corresponding VINCULIN (VINCU) bands. Data represent the mean ± SD of n=2 independent experiments (each in duplicates).

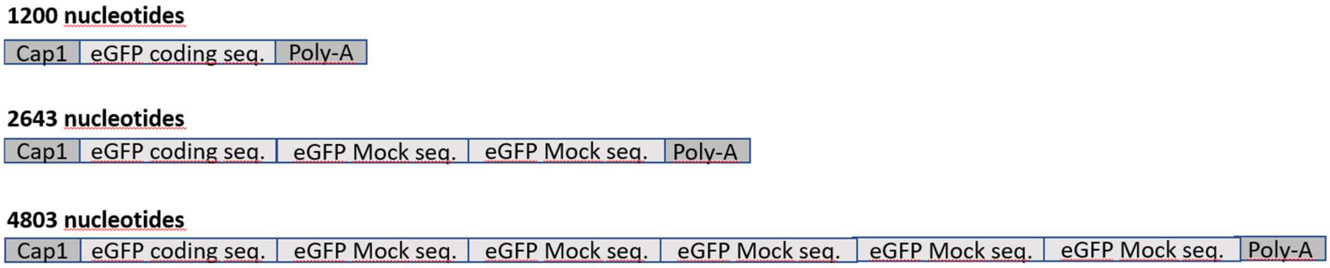

Figure S5: Schematic structure of the mRNA$_{GFP}$ of different lengths

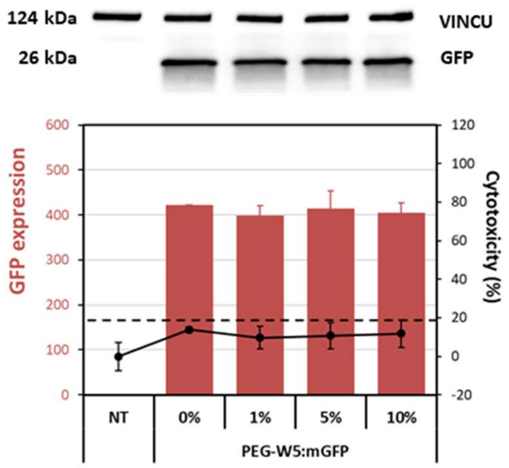

Fig. S6: Effect of the PEGylation of the WRAP5 nanoparticles on the GFP expression activity. WRAP5:mRNA$_{GFP}$ and the indicated PEGylated nanoparticles (CR5, 1 µg mRNA) were transfected to HeLa. GFP expression revealed by Western Blot after a 24 h incubation showed the same GFP expression for the PEGylated WRAP5 nanoparticles as for the naked one. The threshold for non-toxic conditions (= 20%) is visualized by a dashed line. Signal intensities of the GFP bands were normalized to the corresponding Vinculine (VINCU) bands. Data represent the mean ± SD of n=2 independent experiments.

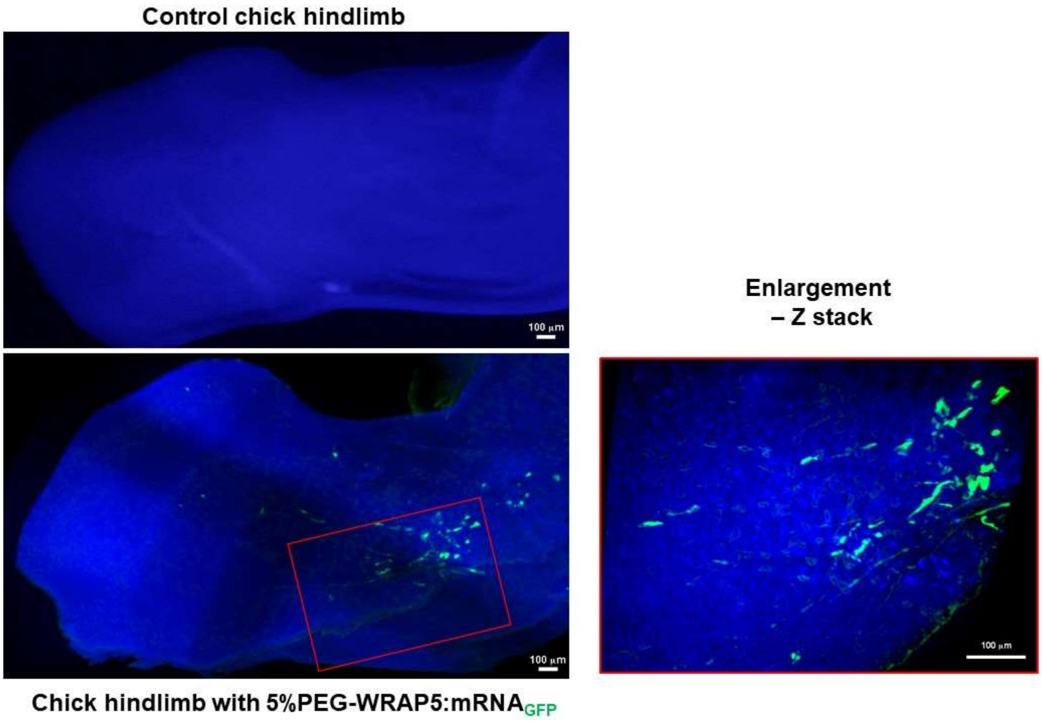

Figure S7: *In vivo* GFP mRNA expression in the chick forelimb. Confocal microscopy analysis after RapiClear® tissue clearing and GFP immunofluorescence staining of E7 whole control (left upper panel) and WRAP5:mRNA$_{GFP}$ (left lower panel) forelimbs. Z-stack acquisition of whole forelimbs (red square) followed by 3D reconstitution for better visualization (right lower panel). TO-PRO-3 for nuclear staining is shown in blue, whereas GFP staining is shown in green. White bars: 100 µm.

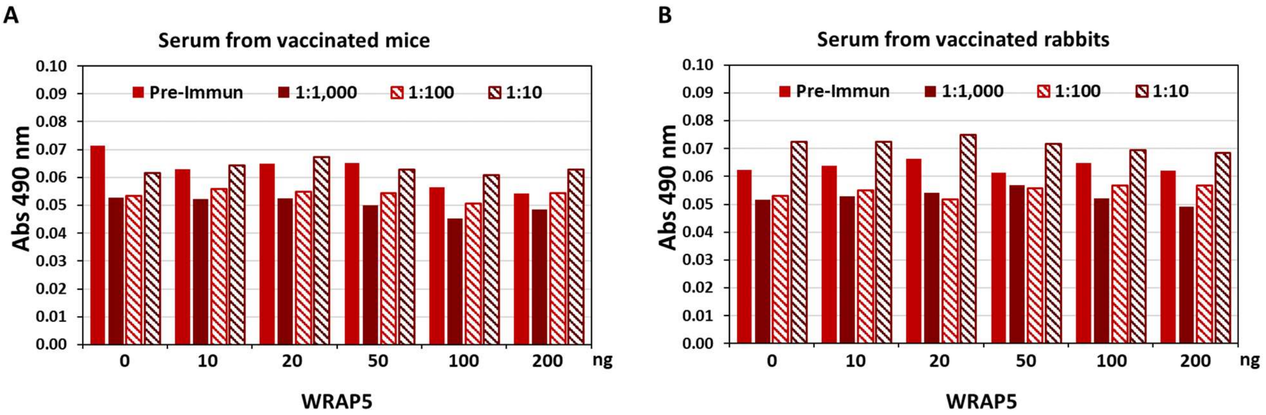

Figure S8: ELISA evaluation of the potential immunological response against the WRAP5 peptide. Different WRAP5 peptide quantities (from 0 to 200 ng) were coated on ELISA plate and incubated with different

dilutions of mice (**A**) or rabbit (**B**) serum (1:10, 1:100 and 1:1,000) and with the pre-immune serum. The potential presence of anti-WRAP5 antibodies was detected with anti-mouse or anti-rabbit secondary HRP-antibodies.
